# 4-Hydr­oxy-2,2,6,6-tetra­methyl­piperidinium chloride–hydroxonium chloride (3/1)

**DOI:** 10.1107/S1600536808002158

**Published:** 2008-01-25

**Authors:** Li Zhang, Peng-Wei Zhang, Xiao-Hui Wang, Li Chen, Qu-Fei Shen

**Affiliations:** a Department of Pharmaceutics, Medical College of the Chinese People’s Armed Police Force, Tianjin 300162, People’s Republic of China; b School of Pharmaceutical Science & Technology, Tianjin University, Tianjin 300072, People’s Republic of China

## Abstract

The crystal structure of the title compound, C_9_H_20_NO^+^·Cl^−^·0.33(H_3_O^+^·Cl^−^), is composed of 4-hydr­oxy-2,2,6,6-tetra­methyl­piperidinium cations, hydroxonium cations and chloride anions, which are connected via O—H⋯O, O—H⋯Cl and N—H⋯Cl hydrogen bonding. The 4-hydr­oxy-2,2,6,6-tetra­methyl­piperidinium cation and one of the two crystallographically independent chloride anions are located on a mirror plane. The hydroxonium cation is located on a threefold axis and the second crystallographically independent chloride anion is located on a sixfold rotoinversion axis. Due to symmetry, the hydroxonium cation is disordered over two positions.
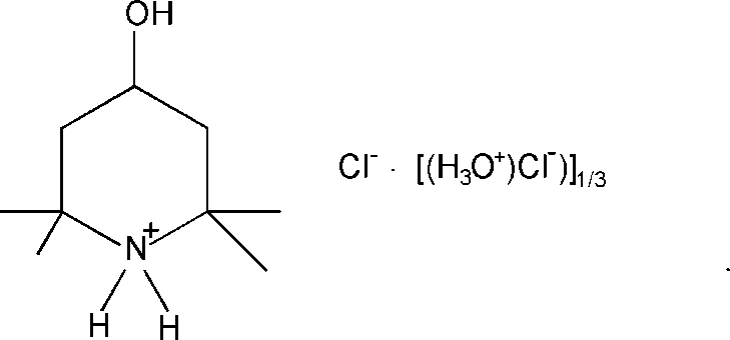

## Experimental

### 

#### Crystal data

C_9_H_20_NO^+^·Cl^−^·0.33(H_3_O^+^·Cl^−^)
*M* *_r_* = 211.87
Hexagonal, 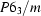

*a* = 13.4460 (19) Å
*c* = 11.528 (2) Å
*V* = 1804.9 (5) Å^3^
*Z* = 6
Mo *K*α radiation
μ = 0.36 mm^−1^
*T* = 113 (2) K
0.10 × 0.10 × 0.04 mm

#### Data collection

Rigaku Saturn diffractometer
Absorption correction: multi-scan (*CrystalClear*; Rigaku/MSC, 2005 ▶) *T*_min_ = 0.968, *T*_max_ = 0.989
14051 measured reflections
1513 independent reflections
1424 reflections with *I* > 2σ(*I*)
*R*_int_ = 0.075

#### Refinement

*R*[*F* ^2^ > 2σ(*F* ^2^)] = 0.051
*wR*(*F* ^2^) = 0.130
*S* = 1.08
1513 reflections
68 parameters
H-atom parameters constrained
Δρ_max_ = 0.33 e Å^−3^
Δρ_min_ = −0.26 e Å^−3^

### 

Data collection: *CrystalClear* (Rigaku/MSC, 2005 ▶); cell refinement: *CrystalClear*; data reduction: *CrystalClear*; program(s) used to solve structure: *SHELXS97* (Sheldrick, 2008 ▶); program(s) used to refine structure: *SHELXL97* (Sheldrick, 2008 ▶); molecular graphics: *SHELXTL* (Sheldrick, 2008 ▶); software used to prepare material for publication: *SHELXTL*.

## Supplementary Material

Crystal structure: contains datablocks I, global. DOI: 10.1107/S1600536808002158/nc2088sup1.cif

Structure factors: contains datablocks I. DOI: 10.1107/S1600536808002158/nc2088Isup2.hkl

Additional supplementary materials:  crystallographic information; 3D view; checkCIF report

## Figures and Tables

**Table 1:** Hydrogen-bond geometry (Å, °)

*D*—H⋯*A*	*D*—H	H⋯*A*	*D*⋯*A*	*D*—H⋯*A*
O1—H1⋯Cl2^i^	0.85	2.15	2.991 (2)	172
N1—H1*B*⋯Cl2^ii^	0.92	2.22	3.139 (2)	175
N1—H1*C*⋯Cl1^iii^	0.92	2.25	3.166 (2)	176
O2—H2⋯O1^iv^	0.85	1.63	2.475 (2)	175

Symmetry codes: (i) 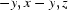; (ii) 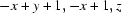; (iii) 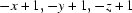; (iv) 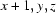.

**Fig. 1.**
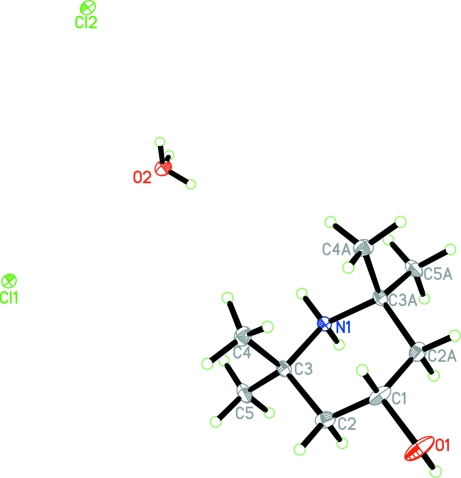
Crystal structure of the title compound with labeling and displacement ellipsoids drawn at the 30% probability level. H atoms are shown as small spheres of arbitrary radii. Symmetry codes: A = x,y,0.5 - z.

## supplementary crystallographic information

### Comment

The title compound was obtained as a byproduct in the synthesis of hindered light stabilizer. For the characterization of this compound a crystal structure analysis was performed. The crystal structure of this compound consists of 4-hydroxy-2,2,6,6-tetramethylpiperidinium cations, hydroxonium cations and chloride anions, all of them located in special positions (Fig. 1). The cations and the anions are connected *via* O—H···O, O—H···Cl and N—H···Cl hydrogen bonding (Table 1). The isolated water oxygen atom, which is located on a 3-fold axis is disordered in two positions because of symmetry. For charge balance it must be protonated. However, the corresponding H atom was clearly located in difference map.

### Experimental

0.25 g(1.6 mmol) of 2,2,6,6-tetramethylpiperidin-4-ol was dissolved in 3.2 ml of hydrochloric acid (3.2 mmol. Colorless crystals of the title compound were obtained by slow evaporation of the solvent.

### Refinement

The C—H and N—H H atoms were positioned with idealized geometry (methyl allowed to rotate but not to tip) and refined isotropic with *U*_iso_(H)=1.2 *U*_eq_(carrier atom; 1.5 for methyl H atoms) using a riding model with C—H = 0.98 - 1.00Å and N—H = 1.00 Å. The O—H H atoms were located in different map, their bond length were set to 0.85Å and afterwards they were refined isotropic (*U*_iso_(H)=1.2*U*_eq_(carrier atom)) using a riding model.

### Crystal data

**Table:**

C_9_H_20_NO^+^·Cl^–^·0.33(H_3_O^+^·Cl^–^)	*Z* = 6
*M**_r_* = 211.87	*F*_000_ = 692
Hexagonal, *P*6_3_/*m*	*D*_x_ = 1.170 Mg m^−^^3^
*a* = 13.4460 (19) Å	Mo *K*α radiation λ = 0.71073 Å
*b* = 13.4460 (19) Å	Cell parameters from 4368 reflections
*c* = 11.528 (2) Å	θ = 1.8–27.9º
α = 90º	µ = 0.36 mm^−^^1^
β = 90º	*T* = 113 (2) K
γ = 120º	Block, colorless
*V* = 1804.9 (5) Å^3^	0.10 × 0.10 × 0.04 mm

### Data collection

**Table:**

Rigaku Saturn diffractometer	1513 independent reflections
Radiation source: rotating anode	1424 reflections with *I* > 2σ(*I*)
Monochromator: confocal	*R*_int_ = 0.075
Detector resolution: 7.31 pixels mm^-1^	θ_max_ = 27.9º
*T* = 113(2) K	θ_min_ = 2.5º
ω scans	*h* = −13→17
Absorption correction: multi-scan(CrystalClear; Rigaku/MSC, 2005)	*k* = −17→16
*T*_min_ = 0.968, *T*_max_ = 0.989	*l* = −14→15
14051 measured reflections	

### Refinement

**Table:**

Refinement on *F*^2^	Secondary atom site location: difference Fourier map
Least-squares matrix: full	Hydrogen site location: inferred from neighbouring sites
*R*[*F*^2^ > 2σ(*F*^2^)] = 0.051	H-atom parameters constrained
*wR*(*F*^2^) = 0.130	*w* = 1/[σ^2^(*F*_o_^2^) + (0.0699*P*)^2^ + 0.5807*P*] where *P* = (*F*_o_^2^ + 2*F*_c_^2^)/3
*S* = 1.08	(Δ/σ)_max_ < 0.001
1513 reflections	Δρ_max_ = 0.33 e Å^−^^3^
68 parameters	Δρ_min_ = −0.25 e Å^−^^3^
Primary atom site location: structure-invariant direct methods	Extinction correction: none

### Special details

**Table:**

Geometry. All e.s.d.'s (except the e.s.d. in the dihedral angle between two l.s. planes) are estimated using the full covariance matrix. The cell e.s.d.'s are taken into account individually in the estimation of e.s.d.'s in distances, angles and torsion angles; correlations between e.s.d.'s in cell parameters are only used when they are defined by crystal symmetry. An approximate (isotropic) treatment of cell e.s.d.'s is used for estimating e.s.d.'s involving l.s. planes.
Refinement. Refinement of *F*^2^ against ALL reflections. The weighted *R*-factor *wR* and goodness of fit *S* are based on *F*^2^, conventional *R*-factors *R* are based on *F*, with *F* set to zero for negative *F*^2^. The threshold expression of *F*^2^ > σ(*F*^2^) is used only for calculating *R*-factors(gt) *etc*. and is not relevant to the choice of reflections for refinement. *R*-factors based on *F*^2^ are statistically about twice as large as those based on *F*, and *R*- factors based on ALL data will be even larger.

### Fractional atomic coordinates and isotropic or equivalent isotropic displacement parameters (Å^2^)

**Table:**

	*x*	*y*	*z*	*U*_iso_*/*U*_eq_	Occ. (<1)
O1	−0.13431 (15)	0.43006 (16)	0.2500	0.0420 (6)	
H1	−0.0986	0.5031	0.2500	0.050*	
N1	0.16134 (16)	0.39830 (16)	0.2500	0.0174 (4)	
H1B	0.2019	0.3598	0.2500	0.021*	
H1C	0.2145	0.4755	0.2500	0.021*	
C1	−0.0587 (2)	0.3833 (2)	0.2500	0.0283 (6)	
H1A	−0.1063	0.2976	0.2500	0.034*	
C2	0.01605 (15)	0.42088 (15)	0.35766 (18)	0.0260 (4)	
H2A	−0.0339	0.3965	0.4271	0.031*	
H2B	0.0625	0.5058	0.3587	0.031*	
C3	0.09668 (15)	0.37155 (15)	0.36468 (16)	0.0213 (4)	
C4	0.03335 (16)	0.24188 (16)	0.38919 (18)	0.0279 (4)	
H4A	−0.0362	0.2037	0.3415	0.042*	
H4B	0.0123	0.2284	0.4714	0.042*	
H4C	0.0836	0.2108	0.3702	0.042*	
C5	0.18849 (18)	0.43315 (17)	0.45708 (17)	0.0302 (4)	
H5A	0.2438	0.4060	0.4542	0.045*	
H5B	0.1523	0.4170	0.5338	0.045*	
H5C	0.2283	0.5161	0.4426	0.045*	
Cl1	0.6667	0.3333	0.7500	0.0219 (3)	
Cl2	0.71701 (5)	0.03169 (5)	0.2500	0.0244 (2)	
O2	0.6667	0.3333	0.3252 (4)	0.0276 (10)	0.50
H2	0.7352	0.3704	0.2999	0.041*	0.50

### Atomic displacement parameters (Å^2^)

**Table:**

	*U*^11^	*U*^22^	*U*^33^	*U*^12^	*U*^13^	*U*^23^
O1	0.0168 (9)	0.0200 (9)	0.091 (2)	0.0103 (8)	0.000	0.000
N1	0.0169 (9)	0.0186 (9)	0.0159 (11)	0.0083 (8)	0.000	0.000
C1	0.0176 (12)	0.0170 (11)	0.052 (2)	0.0096 (9)	0.000	0.000
C2	0.0239 (9)	0.0212 (8)	0.0318 (11)	0.0105 (7)	0.0114 (8)	0.0049 (7)
C3	0.0232 (8)	0.0232 (8)	0.0173 (9)	0.0114 (7)	0.0050 (7)	0.0036 (6)
C4	0.0278 (9)	0.0248 (9)	0.0313 (11)	0.0133 (8)	0.0075 (8)	0.0092 (8)
C5	0.0376 (10)	0.0329 (10)	0.0162 (10)	0.0146 (9)	−0.0007 (8)	0.0022 (8)
Cl1	0.0202 (3)	0.0202 (3)	0.0254 (6)	0.01012 (16)	0.000	0.000
Cl2	0.0251 (3)	0.0203 (3)	0.0295 (4)	0.0125 (2)	0.000	0.000
O2	0.0243 (14)	0.0243 (14)	0.034 (3)	0.0121 (7)	0.000	0.000

### Geometric parameters (Å, °)

**Table:**

O1—C1	1.438 (3)	C2—H2B	0.9900
O1—H1	0.8500	C3—C5	1.524 (3)
N1—C3	1.523 (2)	C3—C4	1.536 (2)
N1—C3^i^	1.523 (2)	C4—H4A	0.9800
N1—H1B	0.9200	C4—H4B	0.9800
N1—H1C	0.9200	C4—H4C	0.9800
C1—C2	1.516 (2)	C5—H5A	0.9800
C1—C2^i^	1.516 (2)	C5—H5B	0.9800
C1—H1A	1.0000	C5—H5C	0.9800
C2—C3	1.530 (2)	O2—O2^i^	1.733 (10)
C2—H2A	0.9900	O2—H2	0.8501
			
C1—O1—H1	113.0	N1—C3—C5	105.47 (14)
C3—N1—C3^i^	120.43 (18)	N1—C3—C2	107.23 (15)
C3—N1—H1B	107.2	C5—C3—C2	111.00 (16)
C3^i^—N1—H1B	107.2	N1—C3—C4	110.77 (15)
C3—N1—H1C	107.2	C5—C3—C4	109.09 (15)
C3^i^—N1—H1C	107.2	C2—C3—C4	113.01 (14)
H1B—N1—H1C	106.9	C3—C4—H4A	109.5
O1—C1—C2	110.50 (13)	C3—C4—H4B	109.5
O1—C1—C2^i^	110.51 (13)	H4A—C4—H4B	109.5
C2—C1—C2^i^	109.9 (2)	C3—C4—H4C	109.5
O1—C1—H1A	108.6	H4A—C4—H4C	109.5
C2—C1—H1A	108.6	H4B—C4—H4C	109.5
C2^i^—C1—H1A	108.6	C3—C5—H5A	109.5
C1—C2—C3	113.23 (16)	C3—C5—H5B	109.5
C1—C2—H2A	108.9	H5A—C5—H5B	109.5
C3—C2—H2A	108.9	C3—C5—H5C	109.5
C1—C2—H2B	108.9	H5A—C5—H5C	109.5
C3—C2—H2B	108.9	H5B—C5—H5C	109.5
H2A—C2—H2B	107.7	O2^i^—O2—H2	69.9
			
O1—C1—C2—C3	178.55 (15)	C3^i^—N1—C3—C4	74.5 (2)
C2^i^—C1—C2—C3	−59.2 (2)	C1—C2—C3—N1	51.7 (2)
C3^i^—N1—C3—C5	−167.57 (14)	C1—C2—C3—C5	166.46 (16)
C3^i^—N1—C3—C2	−49.2 (2)	C1—C2—C3—C4	−70.6 (2)

Symmetry codes: (i) *x*, *y*, −*z*+1/2.

### Hydrogen-bond geometry (Å, °)

**Table:**

*D*—H···*A*	*D*—H	H···*A*	*D*···*A*	*D*—H···*A*
O1—H1···Cl2^ii^	0.85	2.15	2.991 (2)	172
N1—H1B···Cl2^iii^	0.92	2.22	3.139 (2)	175
N1—H1C···Cl1^iv^	0.92	2.25	3.166 (2)	176
O2—H2···O1^v^	0.85	1.63	2.475 (2)	175

Symmetry codes: (ii) −*y*, *x*−*y*, *z*; (iii) −*x*+*y*+1, −*x*+1, *z*; (iv) −*x*+1, −*y*+1, −*z*+1; (v) *x*+1, *y*, *z*.
